# Financial inclusion and improved water usage among households in Ghana

**DOI:** 10.1186/s12889-024-18715-3

**Published:** 2024-05-15

**Authors:** Mustapha Immurana, Kwame Godsway Kisseih, Yakubu Mbanba Ziblilla, Toby Joseph Mathew Kizhakkekara, Micheal Kofi Boachie, Babamu Osman Halidu, Jamal Mohammed, Ibrahim Kaleem, Ayisha Mohammed, Phidelia Theresa Doegah

**Affiliations:** 1 Institute of Health Research, University of Health and Allied Sciences, Ho, Ghana; 2 Christian Health Association of Ghana, Accra, Ghana; 3 Department of Applied Economics, University for Development Studies, Tamale, Ghana; 4 PG Department of Economics, EKNM Government College Elerithattu, Elerithattu (PO), Kasaragod District, Kerala, 671314 India; 5 School of Nursing and Public Health, College of Health Sciences, University of KwaZulu-Natal, Pietermaritzburg, South Africa; 6 Department of Accountancy and Accounting Information Systems, Kumasi Technical University, Kumasi, Ghana; 7 Department of Genera/Liberal Studies, Koforidua Technical University, Koforidua, Ghana; 8 Department of Management Studies, School of Business, Simon Diedong Dombo University of Business and Integrated Development Studies, Wa, Ghana; 9 Akenten Appiah-Menka University of Skills Training and Entrepreneurial Development, Kumasi, Ghana

**Keywords:** Financial inclusion, Use of improved water, Ghana, Health and wellbeing, Households

## Abstract

**Background:**

In Ghana, about 76% of households are at risk of drinking water polluted with faecal matter, hence, poor sanitation and unsafe water are responsible for 80% of all diseases in the country. Given this, some studies have been carried out concerning the factors that determine access and use of improved water among households in Ghana. However, although financial inclusion can make it easy for households to afford and hence, use improved water, it has received very little attention. This study, thus, examines the effect of financial inclusion on the use of improved water among households in Ghana.

**Methods:**

The Ghana Living Standards Survey round 7 (GLSS7) is used as the data source while the binary logit regression is employed as the main empirical estimation technique.

**Results:**

The results show that households with financial inclusion (employing an indicator which has not been disaggregated into formal and informal financial inclusion) have a higher likelihood of using improved water sources relative to those without financial inclusion. The results are robust using formal financial inclusion as well as a combined index of financial inclusion.

**Conclusion:**

Enhancing financial inclusion, especially formal financial inclusion can be utilised as a major policy instrument towards increasing access and use of improved water sources among households in Ghana.

## Introduction

While improved water (water from a source protected from external contamination by its construction) [1] remains an essential good for human development [2], in 2020, estimates showed that 367 million people in the world were drinking unimproved water [3]. In the same year, 16% of the population in Sub-Saharan Africa (SSA) used drinking water sources that were unimproved [3]. The use of contaminated water results in diseases such as cholera, diarrhoea, typhoid and dysentery [4]. Moreover, each year, at the global level, 505,000 diarrhoeal deaths occur due to microbiologically contaminated drinking water [4].

The story in Ghana is not different as about 76% of households are at risk of drinking faeces contaminated water [5]. It is therefore not surprising that 80% of all diseases in Ghana are traceable to poor sanitation and unsafe water [6]. For instance, diarrhoea is regarded as the third most reported health condition at health centers and it accounts for 25% of all under-five mortalities in Ghana [6].

A major factor that could enhance access and use of improved water sources is financial inclusion. Financial inclusion implies sustainable means of making affordable and useful financial services and products easily available to firms, individuals and households in order to help them meet their needs [7, 8]. Thus, given that according to the theory of demand for health, income is a major determinant of demand for health inputs [9] (such as improved water), financial inclusion will improve poverty reduction and income generation [10–12], hence, bolstering the capability of people, especially the poor, to acquire such health inputs. Financial inclusion is thus recognised as a key catalyst in achieving some of the United Nations’ Sustainable Development Goals (SDGs) [8, 13].

Nonetheless, while some empirical studies have been carried out regarding the drivers of access or use of improved water sources in other settings [14–23] and Ghana [2, 24–26], only a handful of studies have tackled the relationship between financial inclusion and access or use of improved water sources [27–30]. Specifically, Pories [29] conducted a study in India and found that financial inclusion that improved access to water at home, led at least one member of a household to direct the time that was previously lost on fetching water towards an economic activity. Similarly, financial inclusion has been found to be positively associated with access to basic drinking water among a sample of African countries [30]. In a related study in Nigeria, informal financial inclusion was found to improve access to water among households [28], while Barenberg [27] found financial inclusion to increase investment in the water sector in India. However, none of the above-mentioned studies that took account of financial inclusion was solely devoted to Ghana albeit the challenges with access and use of improved water in the country, and the possible role financial inclusion could play in addressing these challenges. Moreover, paying attention to financial inclusion in the context of Ghana is very important because the Ghana National Financial Inclusion and Development Strategy (2018–2023) among others, aimed at increasing formal financial inclusion among the adult population from 58% (in 2015) to 85% (in 2023) [31]. In addition, to the best of our knowledge, none of the past studies has considered the roles of both formal and informal financial inclusion in accessing or using improved water. Meanwhile, taking into consideration both formal and informal financial inclusion will help in informing as to which of these forms of financial inclusion should be given much attention in the attempt to increase access and use of improved water.

This study therefore examines the effect of financial inclusion (both formal and informal) on the use of improved water for drinking and general purpose among households in Ghana. By doing so, to the best of our knowledge, the study becomes the first empirical examination of the effect of financial inclusion on the use of improved water sources focused solely on Ghana. The study is also the first empirical attempt to find out the effects of both formal and informal financial inclusion on the use of improved water. The findings of the study therefore highlight whether enhancing financial inclusion can be employed as a major approach towards achieving the United Nations’ [32] SDG 6.1 (equitable and universal access to affordable and safe drinking water for everyone), as well as other SDGs linked to an enhancement in the use of water sources that are improved.

The rest of the paper is organised as follows. The next section presents the methods employed by the study while the third section outlines the results. In the fourth section, we present the discussion of the results while the last section presents the conclusion of the study.

## Methods

### Data

The Ghana Living Standards Survey round 7 (GLSS7) carried out nationwide from October, 2016 to October, 2017 [33] is used as the source of data for this study. We use the GLSS7 because it is the most recent nationwide living standards survey in Ghana. A two-stage stratified sampling technique was employed in carrying out the survey. In the first stage, 1000 Enumeration Areas (EAs) were chosen to form the main sampling units. The EAs were allocated to the 10 hitherto regions in Ghana by employing probability proportional to size, and were subsequently split into urban and rural areas. Via a complete listing of households in the EAs, the sampling units constituting the secondary component of the survey were obtained. In stage two, 15 households, systematically chosen from each of the EAs, resulted in a sample size of 15,000 households. Nonetheless, the survey successfully interviewed 14,009 households [33].

The number of households used in this study is however less than 14,009 because households who use unknown sources of water (other) and those who use sachet or bottled water for both drinking and general purpose are dropped. Households who use sachet or bottled water for both drinking and general purpose are dropped because per the World Health Organization (WHO) and United Nations Children’s Fund (UNICEF) [34] guidelines, for bottled water to be classified as improved, it should be used for only drinking but not for both drinking and general purpose. It must be noted that, although the guidelines do not state sachet water, the authors treated sachet water like bottled water because they are similar. Thus, for instance, bottled water is classified as an improved source of drinking water if there is a different improved water source for general use. Doing so, we are able to classify bottled and sachet water as improved sources of water because they are not being used for drinking and general purpose at the same time.

### Variables and estimation techniques (baseline)

This study uses two dependent variables: (i) source of drinking water for households and (ii) source of water for general use among households. Per the WHO and UNICEF [34] guidelines, the two dependent variables are classified as improved (pipe-borne, public standpipe/tap, borehole/tube well/pump, protected well, rainwater, bottled water, protected spring and sachet water (1)) and unimproved (tanker supply/vendor provided, unprotected spring, unprotected well, river/stream, dugout/dam/pond/canal/ lake (0)).

Financial inclusion is the main independent or explanatory variable, and in our baseline analysis, it is measured by whether the household head possesses a bank account or is a contributor to an informal or formal saving or loan scheme/venture (the main independent variable has two responses: yes or no). The rest of the explanatory variables comprise household size, sex, religion, educational qualification, total household gross income (income), region, residence and age. Sex, religion, education and age are applicable to the household head and the remaining variables are at the household level. For the purpose of this study, we recoded religion and education. Apart from household size, age and income that are continuous, the rest of the explanatory variables are categorical, hence they are handled as dummy variables. Table 1 provides a summary of the measurement of variables.

**Table 1:** Measurement of variables

Variable	Measurement	Coding
Water source (for drinking and general use)	Categorical	Improved water (1), unimproved water (0)
Financial inclusion (baseline measure)	Categorical	Yes (1), no (2)
Sex	Categorical	Male (1), female (2)
Religion	Categorical	No religion (1), Christian (2), Traditionalist/other (3), Islam (6)
Educational qualification	Categorical	None (0), yes (1)
Household size	Continuous	Not applicable
Total household gross income (in Ghana Cedis [GHS])	Continuous	Not applicable
Region	Categorical	Western (1), Central (2), Greater Accra (3), Volta (4), Eastern (5), Ashanti (6), Brong Ahafo (7), Northern (8), Upper East (9), Upper West (10)
Residence	Categorical	Urban (1), rural (2)
Age (in years)	Continuous	Not applicable

Source: Authors, based on the GLSS7

With regard to the statistical estimation techniques, descriptive statistics of the variables are presented using percentages, means, maximum and minimum values, while the Pearson Chi2 is employed to find out the extent of association between the dependent variables and the categorical explanatory variables. However, given that the Pearson Chi2 is a bivariate data analysis technique and does not control for other factors that can affect the dependent variables, the binary logit regression is used for multivariate analysis in order to examine the effect of financial inclusion on the use of improved water while controlling for the rest of the explanatory variables. The binary logit regression is employed since the dependent variables are binary in nature [35].

### Robustness checks

To examine the robustness of our baseline estimates, we break down financial inclusion into informal and formal and find out how they affect the use of improved water for drinking and general purpose. Thus, we compare (i) informal financial inclusion with no financial inclusion, (ii) formal financial inclusion with no financial inclusion and (iii) formal financial inclusion with informal financial inclusion. Informal financial inclusion is measured by having an account in an informal loans and savings venture called ‘susu’. Formal financial inclusion involves the possession of accounts in rural banks, credit unions, formal loans and savings firms, commercial banks and having a mortgage. Both formal and informal financial inclusion are binary in nature (Yes (1), no (0)).

Given the multidimensional nature of financial inclusion [30, 36–38], as another robustness check, we employ the Principal Component Analysis (PCA) technique to create a combined index of financial inclusion and find out how does it affect the use of improved water for drinking and general purpose among the chosen households. The index, which is a continuous variable, is created using the following variables: the possession of accounts in (i) rural banks, (ii) credit unions, (iii) formal loans and savings firms, (iv) commercial banks as well as (v) mobile money, (vi) having a mortgage, utilising (vii) Automated Teller Machine (ATM), (viii) a cheque book, (ix) electronic banking and (x) E-zwich (a card used for financial transaction in Ghana), and xi) involvement in ‘susu’. The binary logit regression is utilised as the estimator for the robustness checks due to the aforementioned reasons.

### Weighting of data

Weighting is applied to the analysis in this study per the recommendation of the Ghana Statistical Service (GSS) and ICF [39], in order to curtail any biasedness resulting from the intricate sampling frame utilised in carrying out the survey (GLSS7), hence making our findings nationally and regionally representative [39]. STATA version 14.0 is used for the statistical analysis in this study.

## Results

The descriptive statistics, bivariate, baseline and robustness regression results (Tables 2, 3, 4, 5, 6 and 7) of the study are presented in this section.

**Table 2:** Descriptive statistics of variables

Variable			%
**Drinking water**			
Unimproved			9.95
Improved			90.05
**Water for general use or purpose**			
Unimproved			12.17
Improved			87.83
**Financial inclusion (baseline measure)**			
Yes			55.46
No			44.54
**Sex**			
Male			66.48
Female			33.52
**Religion**			
No religion			6.17
Christian			73.83
Traditionalist/other			3.79
Islam			16.21
**Educational qualification**			
None			30.37
Yes			69.63
**Region**			
Western			10.50
Central			8.48
Greater Accra			17.00
Volta			7.35
Eastern			11.69
Ashanti			23.27
BrongAhafo			9.50
Northern			6.76
Upper East			3.17
Upper West			2.28
**Residence**			
Urban			56.21
Rural			43.79
	*Mean*	*Min*	*Max*
**Age (in years)**	46.33	15	99
**Total household gross income (in GHS)**	26587.00	0	7723805.00
**Household size**	4.22	1	28

Source: Authors’ computation from GLSS7

**Table 3:** Bivariate analysis of use of improved water and categorical independent variables

Variable	Drinking water			Water for general use		
	Unimproved	Improved	*P*-value	Unimproved	Improved	*P*-value
**Financial inclusion(Baseline)**						
Yes	5.73	94.27	0.000***	7.32	92.68	0.000***
No	15.21	84.79		18.21	81.79	
**Sex**						
Male	11.13	88.87	0.000***	13.27	86.73	0.001***
Female	7.63	92.37		10.01	89.99	
**Religion**						
No religion	16.69	83.31	0.000***	19.92	80.08	0.000***
Christian	8.64	91.36		10.45	89.55	
Traditionalist/other	22.77	77.23		29.25	70.75	
Islam	10.31	89.69		13.05	86.95	
**Educational qualification**						
None	12.42	87.58	0.000***	15.78	84.22	0.000***
Yes	5.57	94.43		6.80	93.20	
**Region**						
Western	14.24	85.76	0.000***	15.53	84.47	0.000***
Central	5.76	94.24		8.12	91.88	
Greater Accra	2.00	98.00		2.23	97.77	
Volta	21.78	78.22		32.10	67.90	
Eastern	20.57	79.43		21.77	78.23	
Ashanti	3.73	96.27		4.67	95.33	
BrongAhafo	8.05	91.95		10.11	89.89	
Northern	25.32	74.68		30.59	69.41	
Upper East	5.12	94.88		8.67	91.33	
Upper West	5.46	94.54		7.76	92.24	
**Residence**						
Urban	2.40	97.60	0.000***	3.56	96.44	0.000***
Rural	19.56	80.44		23.13	76.87	

Source: Authors’ computation from GLSS7;^***^p < 0.01

**Table 4:** The effect of financial inclusion on the use of improved water among households in Ghana

	(1)	(2)
	Improved drinking water	Improved water for general use
**Financial inclusion ((Baseline measure) Ref.: No)**		
Yes	0.454^**^	0.412^**^
	(0.0442)	(0.0293)
**Sex (Ref: Female)**		
Male	-0.316^***^	-0.206^**^
	(0.00461)	(0.0486)
**Religion (Ref: No religion)**		
Christian	0.0211	0.124
	(0.921)	(0.527)
Traditionalist/other	0.0144	0.0229
	(0.966)	(0.938)
Islam	0.0109	-0.0616
	(0.969)	(0.816)
**Educational qualification (Ref: None)**		
Yes	0.448^***^	0.486^***^
	(0.0000486)	(0.00000195)
**Household size**	-0.0558^**^	-0.0701^***^
	(0.0125)	(0.000711)
**Total household gross income (in GHS)**	0.00000151^*^	0.00000262^**^
	(0.0961)	(0.0310)
**Region (Ref: Western)**		
Central	0.897^**^	0.749^**^
	(0.0258)	(0.0190)
Greater Accra	0.534	0.841
	(0.376)	(0.149)
Volta	-0.414	-0.788^***^
	(0.192)	(0.00458)
Eastern	-0.622	-0.510
	(0.163)	(0.211)
Ashanti	1.194^***^	1.097^***^
	(0.00641)	(0.00376)
Brong Ahafo	0.790^**^	0.737^**^
	(0.0266)	(0.0159)
Northern	-0.421	-0.548^*^
	(0.226)	(0.0948)
Upper East	1.572^***^	1.123^***^
	(0.000211)	(0.00161)
Upper West	1.198	1.061
	(0.152)	(0.108)
**Residence (Ref: Urban)**		
Rural	-1.833^***^	-1.628^***^
	(3.90e-09)	(2.97e-10)
**Age (in years)**	-0.00548	-0.00518
	(0.288)	(0.256)
Constant	3.473^***^	2.962^***^
	(1.20e-09)	(3.40e-10)
Observations	9496	9496
No. of strata	20	20
No. of primary sampling units	990	990
F-stat	10.44	13.80
F-stat *P*-value	1.42e-28***	6.12e-39***

Source: Authors’ computation from GLSS7; *p*-values in parentheses;^*^p < 0.1, ^**^p < 0.05, ^***^p < 0.01

**Table 5:** The effects of formal and informal financial inclusion on the use of improved drinking water among households in Ghana

	(1)	(2)	(3)
	**Improved drinking water**	**Improved drinking water**	**Improved drinking water**
**Informal financial inclusion (Ref: No financial inclusion)**	0.348		
	(0.134)		
**Formal financial inclusion (Ref: No financial inclusion)**		0.747^***^	
		(3.32e-08)	
**Sex (Ref: Female)**			
Male	-0.426^***^	-0.355^***^	-0.319^*^
	(0.00363)	(0.00380)	(0.0978)
**Religion (Ref: No religion)**			
Christian	-0.0271	0.0627	0.238
	(0.906)	(0.768)	(0.442)
Traditionalist/other	-0.260	0.00584	0.576
	(0.490)	(0.986)	(0.285)
Islam	0.189	-0.0347	-0.00109
	(0.545)	(0.904)	(0.998)
**Educational qualification (Ref: None)**			
Yes	0.416^***^	0.412^***^	0.404^**^
	(0.00323)	(0.00137)	(0.0362)
**Household size**	-0.0442^*^	-0.0515^**^	-0.0625^*^
	(0.0576)	(0.0396)	(0.0913)
**Total household gross income (in GHS)**	0.000000730	0.000000781	0.00000114
	(0.271)	(0.208)	(0.317)
**Region (Ref: Western)**			
Central	0.664	0.790^*^	1.713^***^
	(0.112)	(0.0509)	(0.00205)
Greater Accra	1.328^*^	0.582	0.377
	(0.0516)	(0.337)	(0.528)
Volta	-0.340	-0.287	-0.259
	(0.346)	(0.371)	(0.490)
Eastern	-0.497	-0.386	-0.588
	(0.172)	(0.258)	(0.304)
Ashanti	0.965^*^	1.172^***^	1.933^***^
	(0.0523)	(0.00713)	(0.000192)
Brong Ahafo	0.854^**^	0.806^**^	0.777^*^
	(0.0369)	(0.0235)	(0.0595)
Northern	-0.586	-0.292	-0.0387
	(0.105)	(0.401)	(0.928)
Upper East	2.294^***^	1.649^***^	1.273^**^
	(0.00000571)	(0.000897)	(0.0125)
Upper West	2.196^***^	1.165	0.438
	(0.000137)	(0.182)	(0.667)
**Residence (Ref: Urban)**			
Rural	-1.684^***^	-1.702^***^	-1.838^***^
	(0.00000530)	(2.86e-09)	(2.33e-08)
**Age (in years)**	-0.00194	-0.00318	-0.0136^*^
	(0.702)	(0.521)	(0.0538)
**Formal financial inclusion (Ref: Informal financial inclusion)**			0.815^*^
			(0.0546)
Constant	3.247^***^	3.184^***^	3.500^***^
	(1.50e-09)	(1.23e-10)	(7.75e-09)
Observations	4332	8213	5639
No. of strata	20	20	20
No. of primary sampling units	929	984	916
F-stat	7.483	10.78	7.879
F-stat *P*-value	3.68e-19***	1.31e-29***	2.24e-20***

Source: Authors’ computation from GLSS7; *p*-values in parentheses; ^*^p < 0.1, ^**^p < 0.05, ^***^p < 0.01

**Table 6:** The effects of formal and informal financial inclusion on the use of improved water for general activities among households in Ghana

	(1)	(2)	(3)
	**Improved water for general use**	**Improved water for general use**	**Improved water for general use**
**Informal financial inclusion (Ref: No financial inclusion)**	0.433^**^		
	(0.0278)		
**Formal financial inclusion (Ref: No financial inclusion)**		0.711^***^	
		(3.74e-09)	
**Sex (Ref: Female)**			
Male	-0.367^***^	-0.289^**^	-0.132
	(0.00859)	(0.0142)	(0.446)
**Religion (Ref: No religion)**			
Christian	0.0729	0.163	0.227
	(0.736)	(0.404)	(0.423)
Traditionalist/other	-0.142	0.0586	0.267
	(0.672)	(0.846)	(0.518)
Islam	0.146	-0.0981	-0.226
	(0.627)	(0.720)	(0.592)
**Educational qualification (Ref: None)**			
Yes	0.401^***^	0.457^***^	0.453^**^
	(0.00179)	(0.000142)	(0.0145)
**Household size**	-0.0532^**^	-0.0655^***^	-0.0817^**^
	(0.0137)	(0.00500)	(0.0197)
**Total household gross income (in GHS)**	0.00000330	0.00000158	0.00000131
	(0.124)	(0.185)	(0.266)
**Region (Ref: Western)**			
Central	0.421	0.710^**^	1.539^***^
	(0.241)	(0.0315)	(0.000245)
Greater Accra	1.060^*^	0.915	0.997
	(0.0888)	(0.117)	(0.112)
Volta	-0.762^**^	-0.590^**^	-0.559
	(0.0145)	(0.0378)	(0.113)
Eastern	-0.517	-0.273	-0.336
	(0.122)	(0.375)	(0.543)
Ashanti	0.753^*^	1.100^***^	1.924^***^
	(0.0853)	(0.00389)	(0.0000285)
Brong Ahafo	0.722^*^	0.773^**^	0.758^**^
	(0.0504)	(0.0122)	(0.0364)
Northern	-0.857^**^	-0.319	-0.139
	(0.0131)	(0.334)	(0.769)
Upper East	1.171^***^	1.177^***^	1.229^***^
	(0.00668)	(0.00174)	(0.00834)
Upper West	1.950^***^	1.072	0.381
	(0.0000611)	(0.129)	(0.637)
**Residence (Ref: Urban)**			
Rural	-1.651^***^	-1.525^***^	-1.501^***^
	(0.000000121)	(6.35e-11)	(0.000000122)
**Age (in years)**	-0.00362	-0.00339	-0.0101
	(0.418)	(0.443)	(0.134)
**Formal financial inclusion (Ref: Informal financial inclusion)**			0.822^**^
			(0.0241)
Constant	3.086^***^	2.725^***^	2.689^***^
	(4.30e-11)	(1.45e-11)	(0.000000311)
Observations	4332	8213	5639
No. of strata	20	20	20
No. of primary sampling units	929	984	916
F-stat	8.871	14.66	11.59
F-stat *P*-value	1.65e-23***	1.69e-41***	7.44e-32***

Source: Authors’ computation from GLSS7; *p*-values in parentheses;^*^p < 0.1, ^**^p < 0.05, ^***^p < 0.01

### Descriptive statistics

In Table 2, we find that 90.1% (majority) and 87.8% (majority) of the sampled households utilise improved water for drinking and general purpose, respectively. Similarly, the greater proportion of the heads of households are males (66.5%), have acquired some form of formal education (69.6%) and are also Christians (73.8%).

With regard to financial inclusion (baseline measure), 55.5% of the heads of households are financially included while 44.5% are not. Descriptive statistics of the rest of the variables can be found in Table 2.

### Bivariate analysis

The findings of the bivariate analysis show statistically significant association between the dependent variables and all the categorical explanatory variables at the 1% level (Table 3). The percentage distributions of the categorical explanatory variables and the use of improved water for drinking and general purpose can be seen in Table 3.

**Table 7:** The effect of a combined index of financial inclusion on using improved water among households in Ghana

	(1)	(2)
	**Improved drinking water**	**Improved water for general use**
**Combined index of financial inclusion**	0.514^***^	0.590^***^
	(0.00385)	(0.000152)
**Sex (Ref: Female)**		
Male	-0.307^***^	-0.205^**^
	(0.00491)	(0.0464)
**Religion (Ref: No religion)**		
Christian	0.0456	0.130
	(0.833)	(0.513)
Traditionalist/other	0.0252	0.0291
	(0.940)	(0.921)
Islam	0.0345	-0.0507
	(0.903)	(0.849)
**Educational qualification (Ref: None)**		
Yes	0.443^***^	0.452^***^
	(0.0000997)	(0.0000135)
**Household size**	-0.0520^**^	-0.0661^***^
	(0.0159)	(0.00105)
**Total household gross income (in GHS)**	0.00000141	0.00000234^*^
	(0.129)	(0.0594)
**Region (Ref: Western)**		
Central	0.868^**^	0.712^**^
	(0.0318)	(0.0259)
Greater Accra	0.526	0.819
	(0.385)	(0.160)
Volta	-0.425	-0.801^***^
	(0.182)	(0.00394)
Eastern	-0.624	-0.513
	(0.160)	(0.209)
Ashanti	1.185^***^	1.088^***^
	(0.00710)	(0.00411)
Brong Ahafo	0.815^**^	0.765^**^
	(0.0229)	(0.0124)
Northern	-0.466	-0.602^*^
	(0.178)	(0.0645)
Upper East	1.551^***^	1.085^***^
	(0.000256)	(0.00231)
Upper West	1.181	1.046
	(0.158)	(0.113)
**Residence (Ref: Urban)**		
Rural	-1.820^***^	-1.608^***^
	(3.04e-09)	(2.94e-10)
**Age (in years)**	-0.00533	-0.00481
	(0.283)	(0.275)
Constant	3.666^***^	3.170^***^
	(3.65e-12)	(3.22e-13)
Observations	9498	9498
No. of strata	20	20
No. of primary sampling units	990	990
F-stat	10.68	14.64
F-stat *P*-value	2.43e-29***	1.89e-41***

Source: Authors’ computation from GLSS7; *p*-values in parentheses; ^*^p < 0.1, ^**^p < 0.05, ^***^p < 0.01

### Baseline regression results

In this sub-section, the results of the baseline multivariate analysis using the binary logit regression are presented (Table 4). In the first model, we present estimates of the association between financial inclusion and the use of improved drinking water while the second model is devoted to the association between financial inclusion and the use of improved water for general activities.

Starting with financial inclusion, the results show that it has a positive statistically significant association with the use of improved water for drinking and general purpose among households in Ghana. Specifically, households with heads who are financially included are found to be more probable to use improved sources of water for drinking (Coefficient: 0.45, *p* < 0.05) and general purpose (Coefficient: 0.41, *p* < 0.05) relative to households whose heads are financially excluded (i.e. have no financial inclusion) (Table 4).

In relation to how sex of household heads affects the use of improved water, we notice that male-headed households have lesser likelihood of using improved sources of water for drinking (Coefficient: -0.32, *p* < 0.01) and general purpose (Coefficient: -0.21, *p* < 0.05) relative to female-headed households (Table 4).

We also find the formal education level of the head of the household to play an important role concerning the use of improved water sources. Specifically, households with formally educated heads are found to be more likely to use improved sources of water for drinking (Coefficient: 0.45, *p* < 0.01) and general purpose (Coefficient: 0.49, *p* < 0.01) as compared to households whose heads are without formal educational qualification (Table 4).

Also, rising household size is found to be linked with a fall in the probability of using improved water for drinking (Coefficient: -0.06, *p* < 0.05) and general purpose (Coefficient: -0.07, *p* < 0.01). In addition, we find an increase in household gross income to be linked with a rise in the probability of using improved water sources for drinking (Coefficient: 0.000002, *p* < 0.10) and general purpose (Coefficient: 0.000003, *p* < 0.05) (Table 4).

Further, the region of residence has a statistically significant association with the use of improved water. Specifically, households in the Central Region (Coefficient: 0.90, *p* < 0.05; Coefficient: 0.75, *p* < 0.05), Ashanti Region (Coefficient: 1.19, *p* < 0.01; Coefficient: 1.10, *p* < 0.01), BrongAhafo Region (Coefficient: 0.79, *p* < 0.05; Coefficient: 0.74, *p* < 0.05) and Upper East Region (Coefficient: 1.57, *p* < 0.01; Coefficient: 1.12, *p* < 0.01) are more likely to use improved sources of water as compared with those in the Western Region^1^. On the contrary, households in the Volta Region (Coefficient: -0.79, *p* < 0.01) and Northern Region (Coefficient: -0.55, *p* < 0.1) are found to be less likely to use improved water sources for general purpose as compared with those in the Western Region (Table 4).

Examining the effect of residential location (rural or urban) of households on using improved water, we find that households in rural areas are less likely to use improved water sources for drinking (Coefficient: -1.83, *p* < 0.01) and general purpose (Coefficient: -1.63, *p* < 0.01) relative to urban dwellers (Table 4).

### Robustness checks results

In the robustness checks, we find that the association between informal financial inclusion (relative to no financial inclusion) and the use of improved drinking water is positive but insignificant (Coefficient: 0.35, *p* > 0.1). However, formal financial inclusion (as compared with no financial inclusion and informal financial inclusion) is found to have positive significant association (Coefficient: 0.75, *p* < 0.01; and Coefficient: 0.82, *p* < 0.1, respectively) with the use of improved drinking water (Table 5).

Moreover,  relative to no financial inclusion, both informal financial inclusion (Coefficient: 0.43, *p* < 0.05) and formal financial inclusion (Coefficient: 0.71, *p* < 0.01) are found to have positive significant relationship with the use of improved water for general purpose. In addition, relative to informal financial inclusion, formal financial inclusion is revealed to have a positive significant relationship with the use of improved water for general purpose (Coefficient: 0.82, *p* < 0.05) (Table 6). The above indicate that our results are robust (with the exception of informal financial inclusion relative to no financial inclusion in the case of using improved drinking water).

The results of the combined index of financial inclusion are not qualitatively different as they show positive significant association with the use of improved water for drinking (Coefficient: 0.51, *p* < 0.01) and general purpose (Coefficient: 0.59, *p* < 0.01) (Table 7).

## Discussion

Our study demonstrates the role of financial inclusion (especially formal financial inclusion) in enhancing the use of improved water among households in Ghana. Similar finding has been revealed among a sample of African countries [30] and in Nigeria [28]. This finding is not farfetched because, financial inclusion has been shown to reduce poverty and provide income [10–12] which would enhance the ability of households to afford improved water sources. Moreover, financial inclusion has been reported to be linked with a reduction in unhealthy behaviours such as sharing of toilet facilities, open defecation and indiscriminate waste disposal among households in Ghana [40, 41].

The finding on financial inclusion points to the importance of achieving or sustaining the targets of the Ghana National Financial Inclusion and Development Strategy (2018–2023) which among others, aimed to increase formal financial inclusion among the adult population from 58% (in 2015) to 85% (in 2023) [31]. These targets can be achieved or sustained by improving financial sector infrastructure and making sure that innovative, responsible and sound financial institutions make available a wide array of quality and affordable financial services and products that are in line with the needs of all Ghanaians [31]. Also, instituting and enforcing legal protection of consumers, ensuring robust dissolution of disputes as well as a higher understanding of the rights of consumers could be of great help [31]. In addition, efforts should be made towards creating awareness among those who are financially excluded about the importance of financial inclusion in enhancing general wellbeing.

The results of male-headed households having a lesser likelihood of using improved sources of water could be due to the less attention paid by men with regard to domestic or household issues [41]. Thus, in Ghana, women are mostly the domestic managers with regard to ensuring access to household facilities such as improved water [41]. Our finding concurs with those of Adams et al. [25] and Agbadi et al. [2].

The association of formal education with higher likelihood of using improved water among households is not surprising because, via formal education, people will get to know the essence of using improved water sources. Moreover, individuals with formal education are more likely to earn higher income due to their skills [42], hence would have the ability to afford improved water. Beyond improved water sources, formal education has been revealed to be connected with higher probability of using other products that improve health among households in Ghana [43–47]. The outcome of the role of formal education is consistent with some past studies [2, 15, 20].

The finding on rising household size being linked with a fall in the probability of using improved water could be due to the fact that, improved water services such as piped-borne water, normally come at a fee, and the fee increases as the number of users increases. Thus, higher household size may be associated with higher user fees, hence, may deter such households from using improved water sources. Our result is similar to that of Dongzagla [26] regarding urban households in Ghana.

We find household income to play a role in using improved water. Thus, rising household income implies households would be capable of buying, maintaining and repairing improved water sources (that are expensive relative to unimproved water sources). Similar outcome regarding the role of household wealth in accessing improved water has been revealed by past studies [16, 26].

There are regional and locational (urban or rural) disparities regarding the use of improved water among households in Ghana. The less likelihood of households in the Northern and Volta Regions to use improved water sources for general activities could be the fact that, as reported by the Ghana Statistical Service [48], they are poor regions relative to the Western Region, hence, they may not be able to afford improved water sources. The findings on households in the Upper East and Ashanti Regions being more likely to use improved water sources are in tandem with those of Adams et al. [25]. Moreover, since rural residents are likely to be poor (relative to urban residents) [40], hence may not be able to afford improved water sources, it is not surprising that they are less likely to use improved water. Also, rural dwellers have been found to be less likely to have access to improved water sources relative to their urban counterparts [2, 25].

## Conclusion

While the use of unimproved water sources is connected with several negative health and economic effects, a number of people in the world as well as in Ghana use unimproved sources of water for drinking and other purpose. This has led to a number of empirical studies regarding the drivers of access and use of improved water sources. Nonetheless, while financial inclusion can be a useful tool towards increasing access and use of improved water, it has received no attention in the Ghanaian context. This study thus provides the first empirical analysis of the association between financial inclusion and the use of improved sources of water for drinking and general purpose among households in Ghana. To do so, we employ data from the GLSS7 while using the binary logit regression as the main estimator. Our findings show that financial inclusion is linked with a higher probability of using improved water sources among households in Ghana. Our findings are robust after separating financial inclusion into informal and formal (with the exception of informal financial inclusion as compared with no financial inclusion in the case of using improved drinking water) as well as using a combined index of financial inclusion. The implication is that in the attempt to increase the use of improved water sources among households in Ghana, focusing on improving financial inclusion can be a very handy tool.

Notwithstanding the above, our study is not without limitation. We focus on only Ghana; hence our findings cannot be extended to other countries. Future studies may therefore consider using more recent cross-sectional datasets on several countries in order to provide results that are generalisable to many countries.

## Data Availability

The data employed by the study can be obtained freely from the website of the Ghana Statistical Service (https://www2.statsghana.gov.gh/nada/index.php/catalog/97/get_microdata).

## Footnotes

1 With regard to the results for region indicated in parentheses above, the first coefficients and *p*-values are for improved drinking water usage while the second coefficients and *p*-values are for using improved water for general activities.
